# Improving quality of life through the routine use of the patient concerns inventory for head and neck cancer patients: a cluster preference randomized controlled trial

**DOI:** 10.1186/s12885-018-4355-0

**Published:** 2018-04-18

**Authors:** Simon N. Rogers, Derek Lowe, Cher Lowies, Seow Tien Yeo, Christine Allmark, Dominic Mcavery, Gerald M. Humphris, Robert Flavel, Cherith Semple, Steven J. Thomas, Anastasios Kanatas

**Affiliations:** 1 Regional Maxillofacial Unit, University Hospital Aintree, Liverpool, UK; 2 Edge Hill University, Liverpool and Evidence-Based Practice Research Centre (EPRC), Faculty of Health and Social Care, Road, L39 4QP, Ormskirk, St Helens, UK; 3 Head and Neck Clinical Trials, University Hospital Aintree, Clinical Sciences Building, Liverpool, UK; 4 Centre for Health Economics and Medicines Evaluation (CHEME), School of Healthcare Sciences, College of Health and Behavioural Sciences (CoHaBS), Bangor University, Ardudwy Building, Normal Site, Bangor, UK; 5 Leeds Teaching Hospitals and St James Institute of Oncology, Leeds Dental Institute and Leeds General Infirmary, Leeds, UK; 6 School of Medicine, Medical & Biological Sciences, North Haugh, St Andrews, UK; 7 Southway, Guildford, Surrey, UK; 8 Macmillan Health and Wellbeing Service, Ulster Hospital, Upper Newtownards Road, Dundonald, Belfast, UK; 9 Oral and Maxillofacial Surgery Department, University, Bristol, Lower Maudlin Street, Bristol, UK; 10 Consultant Regional Maxillofacial Unit, University Hospital Aintree, L9 1AE, Liverpool, UK

**Keywords:** Head and neck Cancer, Patient concerns inventory, Quality of life, Patient reported outcomes, Intervention

## Abstract

**Background:**

The consequences of treatment for Head and Neck cancer (HNC) patients has profound detrimental impacts such as impaired QOL, emotional distress, delayed recovery and frequent use of healthcare. The aim of this trial is to determine if the routine use of the Patients Concerns Inventory (PCI) package in review clinics during the first year following treatment can improve overall quality of life, reduce the social-emotional impact of cancer and reduce levels of distress. Furthermore, we aim to describe the economic costs and benefits of using the PCI.

**Methods:**

This will be a cluster preference randomised control trial with consultants either ‘using’ or ‘not using’ the PCI package at clinic. It will involve two centres Leeds and Liverpool. 416 eligible patients from at least 10 consultant clusters are required to show a clinically meaningful difference in the primary outcome. The primary outcome is the percentage of participants with less than good overall quality of life at the final one-year clinic as measured by the University of Washington QOL questionnaire version 4 (UWQOLv4). Secondary outcomes at one-year are the mean social-emotional subscale (UWQOLv4) score, Distress Thermometer (DT) score ≥ 4, and key health economic measures (QALY-EQ-5D-5 L; CSRI).

**Discussion:**

This trial will provide knowledge on the effectiveness of a consultation intervention package based around the PCI used at routine follow-up clinics following treatment of head and neck cancer with curative intent. If this intervention is (cost) effective for patients, the next step will be to promote wider use of this approach as standard care in clinical practice.

**Trial registration:**

32,382. Clinical Trials Identifier, NCT03086629. Protocol: Version 3.0, 1st July 2017.

## Background

The incidence of Head and neck cancer (HNC) is increasing, the three main sites being oral cavity (mouth), oropharynx (throat) and larynx (voice box) with about 11,000 new cancers in the UK each year http://www.cancerresearchuk.org/about-cancer/mouth-cancer. Treatments such as surgery and chemo-radiotherapy have a detrimental effect on basic functions including speech, swallowing and appearance. These in turn can have a profound negative influence on emotional well-being and social integration http://www.handle-on-qol.com/About.aspx. Patients often do not raise issues of concern in their follow-up consultations and it can be a challenge for clinicians to facilitate this in a busy clinic [1]. Questionnaire prompt lists (QPL) are a means to allow patients to raise their agenda and help focus consultations [2–5]. The Patient Concerns Inventory (PCI-HN) is an item prompt list specific to head and neck cancer [6] http://www.headandneckcancer.co.uk/professionals/patient-concerns-inventory, and differs from many QPLs, which are more general cancer tools [7]. The PCI-HN was designed for routine clinic consultations within the context of NHS financial constraints. It is freely available http://www.patient-concerns-inventory.co.uk and is in the early phases of development for other cancers and chronic conditions. The PCI consists of 56 clinical items, which patients select from before their appointment, to help guide the outpatient consultation through the symptoms and problems that they may experience following their treatment for HNC. It helps to focus the consultation, aid doctor-patient communication, and can assist in signposting patients to other professional for advice and support.

The PCI supports several national initiatives and is set in the context of the national debate about how to bring about more person-centred care [8, 9] and the National Cancer Survivorship Initiative http://www.ncin.org.uk/cancer_type_and_topic_specific_work/topic_specific_work/survivorship which ‘aims to ensure that those living with and beyond cancer get the care and support they need to lead as healthy and active a life as possible, for as long as possible’. In a survey of the British Association of Head and Neck Oncology Nurses (BAHNON), the PCI at that time, was the preferred assessment and the majority (60%) felt, as a head and neck specific tool, it was ‘most appropriate’ [10].

Oncology review clinics are busy and barriers such as time constraints, a medical focus of the consultation, and lack of level 1 evidence of patient benefit from the use of the PCI, prevents its wider implementation. Although pilot work has shown that patients completing the PCI would like to continue to use it in clinic and that it is feasible, [11, 12] clinicians tend to focus on traditional medical aspects. There is evidence that consultations can be improved through clinicians developing skills in detecting and responding to patient distress, thereby improving their patients’ emotional functioning and reducing psychological distress [13, 14]. Preliminary findings around the PCI suggest that its use in clinic allows emotional issues to be discussed more openly - notably fears of recurrence, anxiety and depression [15, 16]. Hence the PCI could help clinician communication with patients in these important areas and consequently impact on how consultations are constructed.

The PCI provides a process by which the patient has repeated opportunities to raise issues they feel are important and that they want to discuss. It can be argued that the routine repeated use of the PCI in follow-up clinics will benefit patients wanting support to speak more openly about problems or concerns e.g. psychosocial causes of symptoms; need for psychosocial help; to seek explanation and reassurance for more physical explanations about their cancer and about the side-effects of treatment. It is postulated that this will have a positive impact on quality of life and emotional distress and be demonstrable by one year following HNC treatment [17]. Thus far, the majority of evidence related to the PCI-HN has been derived from one clinic setting. By conducting a randomised controlled trial (RCT) across multiple consultants, it will be possible to rigorously evaluate if the repeated inclusion of the PCI-HN in routine post-treatment consultations does make a significant and clinically meaningfully difference in patient reported quality of life and distress.

## Methods/design

This is a preference cluster randomised control trial with consultants either ‘using or ‘not using’ the PCI at clinic. 416 HNC eligible patients from at least 10 consultant clusters are required to show a clinically meaningful difference in the primary outcome, that is having less than good overall QOL at the final one-year clinic as measured by the relevant question on the UWQOL-v4 [18].

Before treatment, eligible patients will be asked to consent to participation in the ‘research cohort’. Patients agree to their clinical data being used (Table 1) and to completing research questionnaires before each post-treatment consultation, some of which might be used in their consultation. Completion of all pre-consultation questionnaires including the PCI items will be by computer (desktop, tablet, IPAD). Quality Assurance is by initial training and later booster sessions for consultants and a post consultation survey of those in the PCI arm. Also, in the first six months of the study a random selection of clinic consultations will be taped in order to check how consultants do or do not use the PCI package.

**Table 1:** Schedule for collecting clinical and demographic details

Timepoint		Trial Period	
	Enrolment	Baseline clinic	Follow up clinics
Gender	X		
DOB	X		
IMD 2015	X		
Smoking and Drinking Details		X	X^a^
Living Situation		X	
Employment		X	
Income		X	
Primary Diagnosis (ICD code)	X		
Tumour Site	X		
Treatment Plan	X		
Ethnicity	X		
TNM Stage	X		
Cancer Staging	X		
Histology (SNOMED)	X		
HPV Status	X		
Co-Morbidity	X		
ACE 27	X		

^a^Completion at patient 6 and 12 month study visit

A Steering Group is guiding the research and a joint-site Management group will manage it. Each site will have regular Project Team meetings to review progress. Day to day management issues will be addressed with each unit Lead Researcher. A data manager (based at Aintree R&D) will have overall responsibility for ensuring data quality and integrity. The study will last three years comprising of set-up and piloting, 12 months of recruitment, 15 months of follow-up and analysis, and then write-up and initial dissemination.

**Fig. 1:**
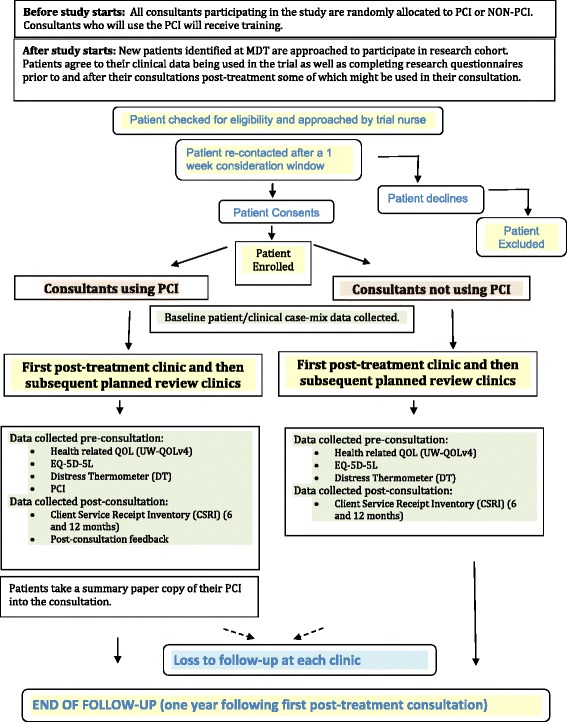
Patient Flow Diagram

In analysis, the two patient groups will be compared after adjusting for relevant case-mix and for effects of patients being within consultant clusters. A summary flowchart of the key features of this trial is shown as Fig. 1.

### Purpose of the study and hypotheses

The main purpose of this three-year research project is to investigate whether incorporating the PCI into routine head and neck cancer (HNC) follow-up consultations improves the overall QOL of patients. The Null hypothesis is that there is no difference between trial groups in the percentage of patients with less than good overall QOL at one year following the first baseline routine clinic post-treatment.

### Participant eligibility

Eligible patients will have a first occurrence of HNC, and be treated curatively (all sites, stage of disease, treatments). To ensure participation of patients with little or no written or spoken English, translation services will be provided as necessary.

Patients treated with palliative intent and patients with a history of previous HNC or recurrence will be excluded from the study. Although the PCI could benefit these patients the primary endpoint of this study is QOL at one year. For reasons of engagement and ethics, patients with a history of cognitive impairment, psychoses or dementia are excluded, as discussed and identified at the staging/treatment decision-making Multi-Professional Team meeting (MDT). Patients who initially are included and treated curatively but who later start receiving treatment with palliative intent will no longer be asked to continue their participation in the research.

### Method of randomisation

Problems of consultant contamination (from switching back and forth from using to not using the PCI package as would be required with conventional randomisation) indicate this should be a cluster RCT, in that consultants are randomised to ‘using or ‘not using’ the PCI at all their trial clinics. The steering group approved a randomisation process incorporating consultant preference; a method reported previously [19]. The aim is to limit the chance occurrence of PCI-sceptic consultants dominating the PCI group and PCI-enthusiastic consultants the non-PCI group. Those with a strong preference are offered their preferred group and those with no preference are randomised. The allocation process was overseen by the medical statistician involved, before any patient recruitment occurred. At Leeds, three of six consultants preferred to be in the PCI group, while the other three consultants had no preference as to group and were all allocated to the non-PCI control group. At Liverpool, three of eight consultants preferred to be in the non-PCI control group. One of the other five consultants was randomly allocated to the non-PCI control group, leaving four to be in the PCI group. Thus, at the two sites, seven consultants were in each arm of the trial.

### Study intervention

Patient completion of the PCI and its inclusion into the regular review clinic consultation within a summary paper output is the ‘intervention’ and is compared to standard out-patient follow-up. The pre-consultation questionnaires and PCI will be used from the first post-treatment clinic (baseline) onwards for one year. The trial will only apply for routine out-patient follow-ups. Completion of all pre-consultation questionnaires and the PCI is by computer (desktop, tablet, IPAD). Assistance (from trained volunteers) will be available to patients as required. Patients of intervention consultants complete the PCI throughout the trial while patients of control consultants do not complete the PCI at all. All study patients will see their consultant surgeon at 6–8 weekly intervals for planned out-patient review. This might be as joint consultation with the oncologist depending on the configuration of the clinic.

While waiting for each consultation the Intervention group patients complete the following:

- Health related QOL (UW-QOLv4)
- EQ-5D-5 L
- Distress Thermometer (DT)
- PCI

**Fig. 2:**
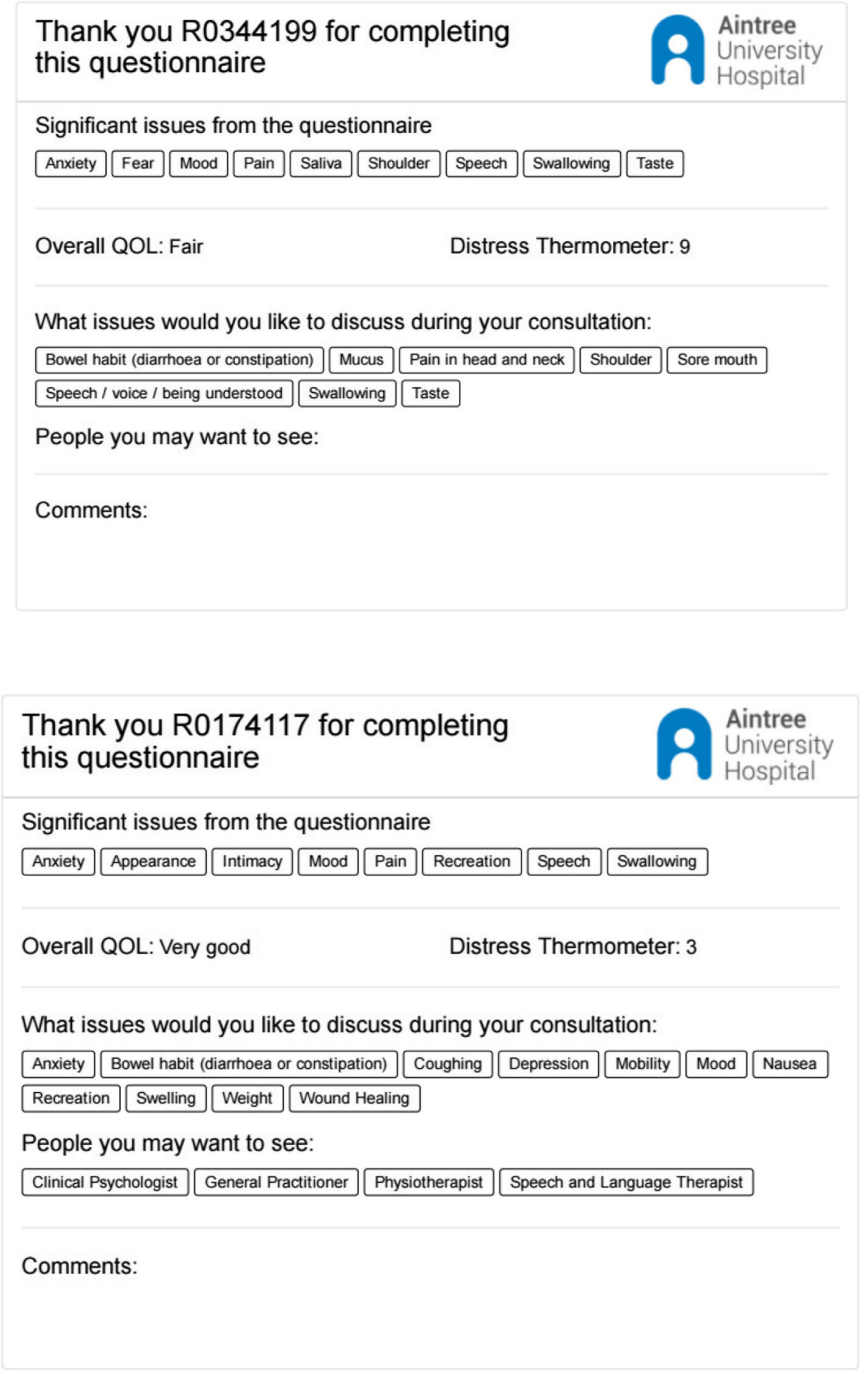
Example of PCI printouts

Intervention patients then take a summary paper output of their data into the clinic consultation (Fig. 2). Post-consultation they will be asked to complete:

- Post-Consultation Patient Feedback about the use of the PCI.
- Client Service Receipt Inventory (CSRI) at 6 and 12 months.

The post consultation data collection will involve self-completion in clinic but either research assisted completion or telephone completion is possible if the patient prefers.

Control patients will complete exactly the same information as intervention patients apart from the PCI and the post consultation feedback on the PCI. They do not take any summary output with them into the consultation. The summary output is a product of the raw inputted data from the patient being run through a software programme that indicates (1) all the items selected from the PCI that the patient wants to discuss (2) those domains from the UWQOL questionnaire for which the patient responses suggest a significant problem or dysfunction (using software algorithms derived from earlier work with the UWQOL [20], (3) the patient’s overall QOL and (4) the Distress Thermometer score. The presence of this summary output during the consultation is the difference in reality between the intervention and control groups as far as the interaction between consultant and patient is concerned.

### Data collection and outcome measures

Unit Clinical Trials Nurses who recruit eligible patients will keep recruitment and clinic attendance logs. The dedicated funded Unit researchers will collect baseline clinical/demographic data either via a baseline clinic questionnaire, with demographic questions chosen as far as possible to match those included in the head and neck 5000 project [21], or by extraction from baseline clinical records. Baseline data will include cancer site, disease severity, HPV status, treatment details, gender, age, deprivation [IMD from post code], smoking, alcohol, and ACE-27 comorbidity. All clinical outcome data will be collected automatically via IPAD at each consultation. A data manager (based at Aintree) will have overall responsibility for ensuring data quality and integrity.

#### Primary outcome measure

The primary outcome measure is overall QOL, specifically the percentage with less than good overall QOL at the final one-year clinic as measured by the single UWQOL-v4 question [18]. The anticipated result in the control group is 30%. The UW-QOLv4 is a commonly used HNC specific HRQOL questionnaire [22, 23] and has been used with HNC patients at the Aintree Regional Head and Neck Unit since 1995. Over 1000 patients have completed over 5000 UW-QOL questionnaires giving the research team considerable experience in analysing and reporting this QOL measure.

#### Secondary outcome measures

1. Mean social-emotional subscale score of UW-QOL
  The Aintree Research team was involved in developing the UW-QOL subscales, and the social-emotional subscale [20] is the mean of 6 domain scores (each 0–100) - anxiety, mood, pain, activity, recreation and shoulder function. The anticipated result in the control group is a mean score of 75.

2. Distress Thermometer (DT) score of 4 or more (range 0 to 10). The anticipated result in the control group is 34%. The Distress Thermometer (DT) is a single item self-report measure and has been used to screen for distress in various cancers [24–27]. A score of four and above denotes significant distress as this correlates with optimal sensitivity and specificity to the Hospital Anxiety Depression Scale [25, 28].

#### Cost-effectiveness

1. Quality-adjusted life year (QALY).
  Quality-adjusted life year (QALY) is used as a summary measure of health benefit for economic evaluation, using the EQ-5D-5 L health index to adjust for patient QOL [29, 30]. QALY is used as a common unit to allow comparisons across different interventions or disease areas [31]. EQ-5D-5 L is a validated generic, health-related, preference-based measure comprising mobility, self-care, usual activities, pain and discomfort, anxiety and depression. These are complemented by a visual analogue scale (VAS) [32], on which patients are asked to indicate their current health from 0 (worst imaginable health) to 100 (best imaginable health) [33].

2. Health service use and costs
  Health service use by participating patients will be collected using a Client Service Receipt Inventory (CSRI). CSRI is a form that is usually administered in an interview setting or by self-completion via postal surveys or at clinics – asking them to recall retrospectively the type and frequency of their contacts with primary and secondary care NHS services. A CSRI form adapted to the study was developed using existing CSRIs, available from the DIRUM open access database http://www.dirum.org, as a reference and guidance with input from study researchers. For this trial, services such as physiotherapist, occupational therapist, social worker and others are included. To translate the service use into costs, unit costs from published sources will be applied to the patients’ self-reported service use data and the mean total cost of care per patient over 12 months will be calculated in each group. CSRI is the most common means of collecting service use data, usually with a short recall period of up to 6 months [34, 35], in health economics studies that require data across a range of health care settings. CSRIs were developed first in the field of mental health economics [36], and a review of their use is published by Ridyard and Hughes [37].

6. Cost of the PCI intervention
  Resources and materials use for the delivery of the PCI intervention will be recorded and costed and the total cost of the PCI intervention will be calculated.

### Sample size calculation

We have used nearly 20 years of accumulated experience with the UW-QOL to estimate a sample size that is pragmatic enough for a trial to be doable, yet able to detect meaningful differences if they exist. In regard to all UWQOL records collected the percentages of patients reporting less than good overall quality of life were relatively similar over different time periods from diagnosis and the expectation for the trial control group was taken as 30% after about 12 months. Cluster randomized trials require larger sample sizes than the individually randomised design because observations on individuals in the same cluster tend to be correlated, thereby reducing the effective sample size. The degree of correlation within consultant clusters, as estimated by the intra-class correlation (ICC) was estimated as barely above zero (6.7e-05) for consultants at Aintree. Assuming a likely control group outcome of 30%, an ICC value of 0.01 for the trial and not wishing to miss a halving in outcome rate, then a total of 312 patients from at least 10 consultants were required. After factoring a likely loss of 15% through patient mortality during the follow-up period and a possible maximum loss of 10% from initial non-consent, this then implied a total of 416 eligible patients needing to be approached for participation to the research. This number would also detect a moderate-sized clinical difference of 10 units (75 Vs. 85) in the mean composite social-emotional subscale score, for which an ICC estimate of 0.025 was obtained for consultants within Aintree. Data from an MD project with 325 HNC patients at Aintree gave an estimated 34% with a Distress Thermometer score ≥ 4, and the trial numbers would be sufficient to detect a halving in this outcome.

### Statistical analysis

As inference will target the individual patient level, analyses will need to adjust for potential clustering in the data. We will report results for each group (PCI, non-PCI) and the estimated effect size from the use of PCI and its precision (95% confidence interval). For the primary outcome, we will report the intra-cluster correlation coefficient to assess the amount of clustering. In reporting results, we will follow the CONSORT statement extension applicable to cluster RCTs. We will use random effects (multi-level) logistic regression methods and will estimate the effect of PCI after making adjustment for relevant case-mix and for clustering effects of patients being within consultant clusters. Only baseline patient factors will be considered as case-mix adjusters and these include age, gender, treatment, overall clinical stage, tumour site and baseline clinic assessment of whether overall UWQOL was less than good (Y/N). A *P* value ≤0.05 is considered statistically significant. Secondary clinical outcomes will be analysed as per protocol.

We will fully cost the delivery of the PCI intervention and associated costs such as training and other materials used. We will use published national average NHS reference consultant costs, accounting for overheads. From an NHS perspective, we will undertake a primary cost-effectiveness analysis of the PCI approach, using the change in % of patients with ‘less than good’ overall QOL between baseline and one-year as the outcome effect, and a subsequent cost-utility analysis using QALY as the outcome effect measured using the EQ-5D-5 L questionnaire. Costs of service use and QALY data will be derived from the CSRI and EQ-5D-5 L questionnaires collected at baseline, 3, 6, 9 and 12 months. The area under the curve method will be used to calculate QALYs, weighting survival by QOL weights obtained from the EQ-5D-5 L. We will compare our findings with unofficial NICE thresholds (ceilings) of £20,000 to £30,000 per QALY. Discounting is unnecessary given the time period. We will account for patient clustering, producing cost-effectiveness planes and acceptability curves (CEACs) to convey to policy makers the probability that PCI approach is cost-effective at different payer thresholds. We will undertake 5000 bootstrapped replications to generate confidence intervals around point estimates. The CSRI also allows us to account for the impact on healthcare service use from intervention participation, important when further rolling out the PCI approach.

### Quality assurance (QA)

Quality Assurance will be ensured by initial training and booster sessions for consultants, together with post consultation patient feedback and audio taping of a number of consultations.

#### Training

There will be a short training programme for staff using the PCI before any patient recruitment. A brief manual/instruction booklet is used to talk through how the PCI should be used in consultations. There will also be two refresher sessions at 4 and 8 months into the trial recruitment phase.

Patients completing the PCI will be asked to complete a post-consultation feedback on paper identified by unique study number and date of clinic; they will be asked to leave this in clinic with the research team; telephone completion of this will also be available. The question is: Did the doctor make reference to the PCI prompt list during the consultation? Response options are ‘Not at all’, ‘A little’, ‘Somewhat’, ‘A great deal’.

Any ‘Not at all’ response will be followed through with the relevant consultant with a view to resolving the issue for future clinics conducted.

#### Fidelity

In the first months of the study a random selection of clinic consultations will be taped. The additional burden of taping is an argument for focusing on the set-up period in order to check how consultants do or do not use the PCI. The tapes will allow a check on if and how the PCI print out is being used and it will allow for a check for contamination in the non-PCI group. It would be expected that between 3 and 6 months into the study, two clinics from each consultant would be taped.

### Management and governance

The trial will be guided by the Steering Group, meeting during the set-up and six-monthly thereafter to ensure progress towards reaching the study’s purpose and to give oversight regarding research governance. Its’ membership includes an independent chairman, at least two other independent members, the two Unit Lead Investigators, Trial Coordinator (Full Time), Research Practitioner (Part-Time), Medical Statistician, Health Economist, IT/data management representation and a patient representative from each Unit.

There will be joint-Unit management group meetings every three months, membership comprising the two Unit Lead Investigators, dedicated funded researchers, IT/data management representation and a patient representative from each Unit. Statistical and Economic representation as required. Within Unit, there will be monthly Project Team meetings, membership comprising Unit Lead investigator, dedicated funded researchers, Clinical Trials Nurse(s), and patient representative. Day to day management issues will be addressed by Unit Researchers and escalate to the Unit Lead Investigator.

## Discussion

There is growing evidence that enhanced symptom monitoring during routine cancer care using patient-reported outcomes benefits patients in respect to HRQOL and survival [38]. The premise of this trial is that the PCI can be integrated into routine clinical consultations with minimal cost implication as the doctor-patient interaction will be more time efficient and facilitate appropriate and targeted multi-professional referrals. The item prompt list approach of the PCI should have direct benefit for the participants. A key issue limiting successful implementation of patient reported outcomes in clinical practice is clinicians’ lack of knowledge on how to effectively utilise PROs data in their clinical encounters [39]. Hence, for this trial there is an educational component and training around the use of the PCI. Also, the patient feedback and analysis of taped consultations will help underpin the evidence related to use of the PCI in the consultation. From this material, it would be possible in the future to develop a more robust training package, informed from the lessons learnt from this trial. In addition, the need for clear system guidelines built into how to most effectively use the PCI for the clinician, the patient and other members of the multi-professional team is recognised [40]. The findings from this trial will inform the development of a PCI manual both for patients and professionals.

The collection of the data in both arms of the trial by touch screen computer-assisted technology (IPAD) has distinct advantages in terms of data capture. With advances in digital health it could be expected that this approach would become regularly employed. Touch screen health-related QOL data collection can be used for scientific documentation as well as in clinical settings [41]. For the purpose of the trial the computer system has been transferred from Aintree to the other sites. This has not been as straightforward as expected. This has caused delays in the use of the IPADs in the other clinics. After completion of this trial, in order to support wider adoption of the PCI approach to patient care, progress is being made in respect to a cloud based platform which should be more readily accessible and easier to use than the current system.

The use of the PCI is a form of intervention in clinic by the consultant. There are other intervention trials that focus to improve function and wellbeing in patients with head and neck cancer. Hansson and colleagues [42] compare a person-centred care intervention in terms of health-related quality of life, disease-specific symptoms or problems, with traditional care as a control group for patients with head and neck cancer. Another trial by van der Hout and co-workers [43] is testing the efficacy, cost-utility and reach of an eHealth self-management application ‘Oncokompas’ to obtain optimal supportive care. Both trials explore different tools in a different context to the PCI in this trial. There are many different ways to help enable patients to recover from head and neck cancer, and the possibility of having several evidence based interventions can only help to improve patient’s outcomes and allow centres to select the most appropriate intervention with their healthcare environment. This study has QOL as the primary outcome. This reflects the importance QOL has in terms of outcome following HNC. Also, given the inherent difficulties in QOL evaluation, such as adaptation, response shift, limitations in questionnaire wording, scaling and scoring, it demonstrates the potential power of the PCI to impact positively in patient care. A positive finding from this research will not only serve to promote wider use of the PCI in HNC, but also accelerate the development, piloting and introduction of the PCI in other cancers and chronic conditions. Level 1 evidence as to the benefits of the PCI in HNC care will help drive up standards of care. This research will add substantially to the evidence supporting the use of question prompt lists in NHS practice.

## Abbreviations

ACE-27: Adult Co-Morbidity Evaluation 27
AUH: Aintree University Hospital
BAHNON: British Association of Head and Neck Oncology Nurses
CEAC: cost-effectiveness planes and acceptability curves
CHEME: Centre for Health Economics and Medicines Evaluation
CoHaBS: College of Health and Behavioural Sciences
CONSORT: Consolidated standards of reporting trials
CSRI: Client Service Receipt Inventory (CSRI)
DIRUM: Database of Instruments for Resource Use Measurement
DT: Distress Thermometer
EHU: Edge Hill University
EPRC: Evidence based Practice Research Centre
EQ-5D-5 L: EuroQol 5 dimension questionnaire
HNC: Head and Neck Cancer
HPV: Herpes Papilloma Virus
HRQOL: Health Related Quality of Life
ICC: Intra-Class Correlation
IMD: Index of Multiple Deprivation
MDT: Multi-Professional Team
NICE: National Institute for Health and Care Excellence
PCI: Patients Concerns Inventory
PCI-HN: Patient Concerns Inventory-Head Neck
PROs: Patient Reported Outcomes
QA: Quality Assurance
QALY: Quality-Adjusted Life Year
QOL: Quality of Life
QPL: Questionnaire prompt lists
R&D: Research and Development
RCT: Randomised Controlled Trial
UWQOL: University of Washington QOL questionnaire
UWQOLv4: University of Washington QOL questionnaire version 4
VAS: Visual Analogue Scale
